# Association between shortened dental configurations and health outcomes: a scoping review

**DOI:** 10.1186/s12903-023-03714-4

**Published:** 2024-01-19

**Authors:** Fernanda Lamounier Campos, Lorrany Gabriela Rodrigues, Julya Ribeiro Campos, Gabriela Aparecida Caldeira Rhodes, Gabrielli Flores Morais, Loliza Luiz Figueiredo Houri Chalub, Raquel Conceição Ferreira

**Affiliations:** 1 Universidade Federal de Minas Gerais, Belo Horizonte, Minas Gerais Brazil; 2 Faculdade Ciências Médicas de Minas Gerais, Belo Horizonte, Brazil; 3 School of Dentistry, Universidade Federal de Minas Gerais, Av. Presidente Antonio Carlos, 6627, Belo Horizonte, Minas Gerais 31270-901 Brazil; 4 Department of Community and Preventive Dentistry, School of Dentistry, Universidade Federal de Minas Gerais, Av. Presidente Antonio Carlos, 6627, Belo Horizonte, Minas Gerais Brazil

**Keywords:** Health, Oral health, Quality of life, Review, Dental arch, Patient satisfaction

## Abstract

This study mapped definitions of shortened dental configurations and health outcomes employed in association studies. A scoping review was conducted using the PubMed/Medline, Scopus, Web of Science, SciELO and Cochrane databases. Two trained researchers selected studies and extracted data. Studies that investigated the association between shortened dental configurations (exposure) and person-centered outcomes (general and oral health) related to health behavior (dietary patterns). Clinical outcomes were classified according to the International Classification of Functioning, Disability and Health and the International Classification of Diseases of the World Health Organization (WHO). Shortened dental configurations were defined as at least 20/21 teeth or the position of the teeth, including esthetics, dental occlusion and periodontal status (Eichner Index, Shortened Dental Arches, Functional Dentition Classification System, Posterior Occluding Pairs, Functional Tooth Units). The initial search resulted in 12,525 records in English, Portuguese and Spanish, 432 of which addressed the association of interest. General health (*n* = 203) and oral health (*n* = 201) were addressed in a similar number of studies. Most outcomes were related to general health (*n* = 184), the most frequent of which were endocrine, nutritional or metabolic diseases (*n* = 57) and mental functions (*n* = 26). Person-centered measures were addressed in 153 studies, most of which were about oral health and oral health-related quality of life (*n* = 62). Oral health outcomes were predominantly related to intake functions (*n* = 44) and diseases or disorders of the orofacial complex (*n* = 24). Dietary patterns (*n* = 43) and mortality (*n* = 38) were also studied. The cross-sectional design (*n* = 257) and non-probabilistic sampling (*n* = 218) were more frequent. The shortened dental configurations defined by the WHO were the most frequent in the studies (*n* = 206). The effects of shortened dental configurations have been investigated mainly in relation to endocrine, nutritional or metabolic diseases and measures of oral health-related quality of life. The findings point to a diversity of health outcomes assessed and substantial methodological variability.

**Supplementary Information:**

The online version contains supplementary material available at 10.1186/s12903-023-03714-4.

## Background

Oral health should be measured from a contextualized, theoretically informed, multidimensional perspective beyond commonly used clinical indices and socio-dental measures [1–5]. Indeed, oral health is defined as multifaceted and includes the capacity to speak, smile, smell, savor, touch, chew, swallow and transmit a variety of emotions through facial expressions with confidence and without pain, discomfort or diseases of the craniofacial complex. It is a fundamental component of health as well as physical and mental wellbeing that reflects physiological, social and psychological attributes that are essential to quality of life (QoL) [6].

This concept is aligned with functioning, which is a dynamic interaction between one’s health condition, environmental factors and personal factors [7]. Thus, the assessment of health status and the results of oral health treatment as well as decisions related to plans and care require both clinical and person-centered measures, valuing the presence/absence of a disease/health condition, but also the perspective, experience, social well being and psychological wellbeing of individuals [8]. Person-centered oral health measures include one’s self-perception about one’s health, measures of oral-health-related quality of life (OHRQoL) and satisfaction with one’s mouth/teeth [9].

Person-centered oral health measures have contributed to the understanding of the effects of tooth loss in life and are considered in the study of different dental configurations [8, 10]. Individuals with shortened dental configurations do not have worse OHRQoL compared to those with more complete dentitions even in the absence of dental prostheses [8, 10–12]. Investigations have also assessed the effect of shortened dental configurations on aspects of functioning related to chewing, diet and food selection [13–15].

With shortened dental configurations, the condition that ensures oral functions is denominated functional dentition (FD). Different definitions of FD have been discussed in the literature. Shortened dental arches (SDA) is a dental configuration with the preservation of the anterior teeth and premolars [16]. The World Health Organization (WHO) adopted the retention of 20 functional natural teeth throughout life without requiring a prosthesis as part of its oral health goals [17]. This number was increased to 21 teeth in 2003 [18]. However, assessing the function of dentitions by the presence of occluding pairs could be more consonant with the status of dentitions than simply counting the number of teeth [19]. The Eichner classification is widely used to represent different occluding pairs considering natural or restored dental contacts between the maxilla and mandible in bilateral areas of premolars and molars [20, 21]. Other studies in the literature have also incorporated occluding pairs with the aim of establishing a broader definition of FD than ensures sufficient oral function considering the number of natural teeth, type of teeth present and number of posterior occluding pairs (POPs) [22] as well as the assessment of the periodontal status of the sextants [23].

In the scenario, the literature offers a growing number of studies on shortened dental configurations associated with different clinical outcomes related to general health [24, 25] and oral health [26, 27], person-centered measures [13–15, 28] related to health behavior (dietary pattern) [29, 30] and mortality [31–33]. Considering the multifaceted nature of oral health, with effects on overall quality of life and wellbeing [6], advances are needed in the design of studies that incorporate oral health in the more comprehensive concept of health. Likewise, the potential of the International Classification of Functioning, Disability and Health (ICF) has recently been discussed for the development of oral health indicators and as a theoretical model that enables describing oral functions on both the physiological and social levels by considering personal and environmental characteristics [34]. It is therefore relevant to identify what definitions of shortened dental configurations and health outcomes have been considered in the literature. Such mapping could demonstrate the comprehensiveness of the literature with regards to the study of shortened dental configurations and health outcomes, describe the types of studies, concepts and methodological approaches employed to operationalize the assessment of functional dentition and summarize the available evidence that could guide future studies based on theoretical models and the multidimensional concept of oral health [7]. Thus, the aim of this scoping review was to map what definitions of shortened dental configurations and health outcomes are employed in association studies.

## Methods

The present scoping review [35, 36] was reported following the Preferred Reporting Items for Systematic Reviews and Meta-Analyses for Scoping Reviews (PRISMA-ScR) [37].

### Research question

The following was the research question: What types of definitions for shortened dental configurations have been considered to investigate associations with health outcomes and what are these outcomes?

From the results found in the data extraction of the articles, categories were defined for the shortened dental configurations and for the outcomes studied in the literature. Shortened dental configurations (exposure variable, determinant, predictor, etc.) were based on the count or position of the teeth present in the oral cavity considering aspects related to esthetics, dental occlusion and/or periodontal status. Definitions with a cutoff point of 20/21 teeth based on the WHO [18], the Eichner Index [20], SDA [38], Functional Dentition Classification System [9, 22, 23, 39–42], count of functional tooth units [43, 44] and count of dental occluding pairs [45, 46] were considered. Moreover, the “other definitions” category grouped dental configurations that considered specific types of teeth present or missing (anterior and/or posterior) [47], that defined different cutoff points from those of the WHO concept (such as the presence of 10 maxillary teeth and six mandibular teeth) [48] or that were based on the unilateral or bilateral absence of posterior teeth [49]. The same study could have used more than one definition of reduced tooth configuration (for example, WHO and occluding pairs) (see Additional file 1).

The health outcomes (response variable) were clinical (related to general or oral health), considering the presence of diseases/adverse conditions or disabilities or functional losses, person-centered (related to general and oral health), health behavior (dietary patterns) and mortality. Clinical health outcomes related to general and oral health were classified according to the International Classification of Functioning, Disability and Health (ICF) [7] when referring to disability or functional aspects (body functions and structures) or activities and participation [7]. The WHO International Classification of Diseases (ICD-11) [50] was employed when the outcome was the presence of diseases/adverse conditions, symptoms, signs or clinical findings not classified by the ICF. For outcomes classified as nutritional disorders by the ICD-11, those associated with obesity, body measures such as waist circumference, body mass index (BMI), weight loss, nutritional status, measures by parameters such as albumin level or by instruments such as the Mini Nutritional Assessment, which classifies participants as well-nourished, at risk of malnutrition or malnourished, were considered. Regarding chewing, the clinical outcomes were the results of objective exams (chewing efficiency/performance) [51], which commonly assess the distribution of the size of food particles after a given number of chewing cycles [52].

Person-centered outcomes (response variables) were assessed using subjective measures based on the report/perception of the person. General life or general health and oral health-related measures were classified. Chewing-related outcomes were results related to chewing function assessed subjectively through questionnaires (chewing ability).

Outcomes related to health behavior were dietary patterns and included nutrient, fiber, vegetable and fruit intake based on the 24-h dietary recall/Healthy Eating Index/ Food Frequency Questionnaire, aspects related to food selectivity and consistency and the habit of avoiding certain foods.

The mortality outcome was extracted as defined in the original article without classification a posteriori of the cause of death according to the ICD-11. A single study could have more than one outcome and may have classified it in more than one category.

### Eligibility criteria

Observational and intervention studies published in Spanish, English and Portuguese that investigated the association between shortened dental configuration and health outcomes were included. For the PubMed/Medline database, the “humans” filter and the age filter from 13 years to 80+ were used. As an inclusion criterion, the studies should consider natural teeth for the definition of shortened dental configurations. Health outcomes should be related to general and/or oral health, both clinical and person-centered (measures reported by the patient/participant), health behavior (dietary patterns) or mortality.

Intervention studies that assessed prosthetic treatment modalities and any type of study that did not include the shortened dental configuration as the exposure variable were excluded. Studies in which the exposure variable was based on tooth loss both without any functioning criteria (number of teeth present or missing or severe tooth loss (cutoff point: nine teeth) were also excluded.

### Information sources and search

Searches for relevant articles were performed in the PubMed/Medline, Scopus, Web of Science, SciELO and Cochrane databases in October 2019 (Initial search) and updated in October 2023 (updating the search performed through the initial search strategy) (Table 1).

**Table 1:** Details of the search strategy used in the databases Pubmed Medline, Scopus, Web of Science, Scielo e Cochrane

*PubMed/Medline*	(“dentition status”) OR “functional dentition”) OR “inadequate dentition”) OR “adequate dentition”) OR “shortened dental arch”) OR “shortened dental arches”) OR “occluding pairs”) OR “occlusal pairs”) OR “functional tooth units”) OR “20 + teeth”) OR “21 teeth”) OR “20 teeth”) OR “number of teeth”) OR “natural teeth”) OR “number of natural teeth”) OR “intact natural dentition”) OR “teeth occlusion”) OR “hierarchical system”) OR “reduced dentitions” Filters activated: Humans, Adult: 19+ years, Adult: 19–44 years, Aged: 65+ years, Middle Aged + Aged: 45+ years, Middle Aged: 45–64 years, 80 and over: 80+ years, Young Adult: 19–24 years, Adolescent: 13–18 years.
*Web of Science*	(TS = (“dentition status”) OR TS = (“functional dentition”) OR TS = (“inadequate dentition”) OR TS = (“adequate dentition”) OR TS = (“shortened dental arch”) OR TS = (“shortened dental arches”) OR TS = (“occluding pairs”) OR TS = (“occlusal pairs”) OR TS = (“functional tooth units”) OR TS = (“hierarchical system”) OR TS = (“20 + teeth”) OR TS = (“21 teeth”) OR TS = (“20 teeth”) OR TS = (“number of teeth”) OR TS = (“natural teeth”) OR TS = (“number of natural teeth”) OR TS = (“intact natural dentition”) OR TS = (“teeth occlusion”) OR TS = (“reduced dentitions”)) AND IDIOMA: (English OR Portuguese OR Spanish) AND TIPOS DE DOCUMENTO: (Article OR Proceedings Paper) Tempo estipulado: Todos os anos. Índices: SCI-EXPANDED, SSCI, A&HCI, CPCI-S, CPCI-SSH, ESCI.
*Scopus*	(ALL (“dentition status”) OR ALL (“functional dentition”) OR ALL(“inadequate dentition”) OR ALL (“adequate dentition”) OR ALL (“shortened dental arch”) OR ALL (“shortened dental arches”) OR ALL (“occluding pairs”) OR ALL (“occlusal pairs”) OR ALL (“functional tooth units”) OR ALL (“hierarchical system”) OR ALL (“20 + teeth”) OR ALL (“21 teeth”) OR ALL (“20 teeth”) OR ALL (“number of teeth”) OR ALL (“natural teeth”) OR ALL (“number of natural teeth”) OR ALL (“intact natural dentition”) OR ALL (“teeth occlusion”) OR ALL (“reduced dentitions”) AND ALL (“oral health”) AND (LIMIT-TO(DOCTYPE,"ar”)) AND (LIMIT-TO (SUBJAREA,"DENT”)) AND (LIMIT-TO (LANGUAGE, “English”) OR LIMIT-TO (LANGUAGE,"Spanish”) OR LIMIT-TO (LANGUAGE, “Portuguese”)
*Scielo*	(“dentition status”) OR (“functional dentition”) OR (“inadequate dentition”) OR (“adequate dentition”) OR (“shortened dental arch”) OR (“shortened dental arches”) OR (“occluding pairs”) OR (“occlusal pairs”) OR (“functional tooth units”) OR (“hierarchical system”) OR (“20 + teeth”) OR (“21 teeth”) OR (“20 teeth”) OR (“number of teeth”) OR (“natural teeth”) OR (“number of natural teeth”) OR (“intact natural dentition”) OR (“teeth occlusion”) OR (“reduced dentitions”) filtro para idioma: espanhol, inglês e português
*Cochrane*	(“dentition status”) OR (“functional dentition”) OR (“inadequate dentition”) OR (“adequate dentition”) OR (“shortened dental arch”) OR (“shortened dental arches”) OR (“occluding pairs”) OR (“occlusal pairs”) OR (“functional tooth units”) OR (“hierarchical system”) OR (“20 + teeth”) OR (“21 teeth”) OR (“20 teeth”) OR (“number of teeth”) OR (“natural teeth”) OR (“number of natural teeth”) OR (“intact natural dentition”) OR (“teeth occlusion”) OR (“reduced dentitions”)”

A broad search was performed with no restrictions regarding period or type of study, using terms/descriptors referring only to the shortened dental configuration to retrieve articles with different health outcomes. The search results were exported to EndNote X9® (Clarivate Analytics) for reference management and the removal of duplicates.

### Study selection, data collection

The search strategy was constructed by two researchers. The articles identified were selected independently by two previously trained researchers who also performed the data extraction. Readings were made of the titles and abstract to identify those eligible for review. In cases of doubt regarding the inclusion of an article in this step of the selection process, the reviewers performed full-text readings. The reviewers then independently classified the studies with regards to the reasons for exclusion. Divergences of opinion were resolved by discussion and consensus. In case of doubt, a discussion was held with two experienced researchers in epidemiological studies. After the confirmation of the selection, full-text readings were performed of all articles. This approach was applied in all steps of the selection process.

### Data items and synthesis of results

Items for data extraction were organized on spreadsheets in Excel and defined during the training of the reviewers, which consisted of reading 10% of full texts identified and extracting the data. This training was carried out before starting data extraction of the total articles. After completing the spreadsheet with the extracted data, meetings were held to discuss possible doubts and check the extraction by the two experienced researchers. The following data were extracted: complete reference, year and setting of study, study design, objectives, sample size, sample recruitment setting, age range of sample, type of sampling (probabilistic or non-probabilistic), form of measuring shortened dental configuration (clinical examination or self-report measure), concept of shortened dental configuration study, health outcome, outcome assessment method, statistical analysis employed (type of analysis and adjustment of association investigated by covariables) and statistical significance of the association between the shortened dental configuration and health outcome. Information on the study design was extracted in accordance with what was originally recorded in the article. When not informed, the reviewers did not define the design.

### Description of studies

The absolute frequency of the studies was obtained according to the study setting and a map was created using Microsoft Power BI®. Diameters of the circles represent the frequency of studies in each location. The absolute and relative frequency of studies according to the shortened dental configuration employed were obtained for each year, enabling the demonstration of the use of configurations over time. The frequency of studies according to the method used for the assessment of the dental configuration was also determined.

The count of studies with clinical health outcomes (general and oral health) and health behaviors as well as studies with the mortality outcome was performed considering the classification of the ICF and ICD. The frequency of studies that assessed patient-centered outcomes was also determined. Tables demonstrated the responses adopted in the studies. Instruments for assessing patient-centered outcomes were selected.

Next, the frequency of studies according to the types of shortened dental configurations for the outcomes assessed was determined and demonstrated in bubble charts using Microsoft Excel®. The number of studies identified was plotted on the Y axis and the types of shortened dental configurations were plotted on the X axis. Health outcomes were represented in different bubble colors according to each category. The diameter of each circle represented the relative frequency of the use of a particular shortened dental configuration as exposure considering the total of studies conducted with each of the health outcomes analyzed. The distribution of studies according to the types of designs was also determined considering the type of shortened dental configuration and health outcomes analyzed and the quantity of studies in which the association investigated was statistically significant.

### Protocol and registration

The protocol was registered by the protocols.io (DOI: 10.17504/protocols.io.q26g7yjn1gwz/v1).

## Results

**Fig. 1:**
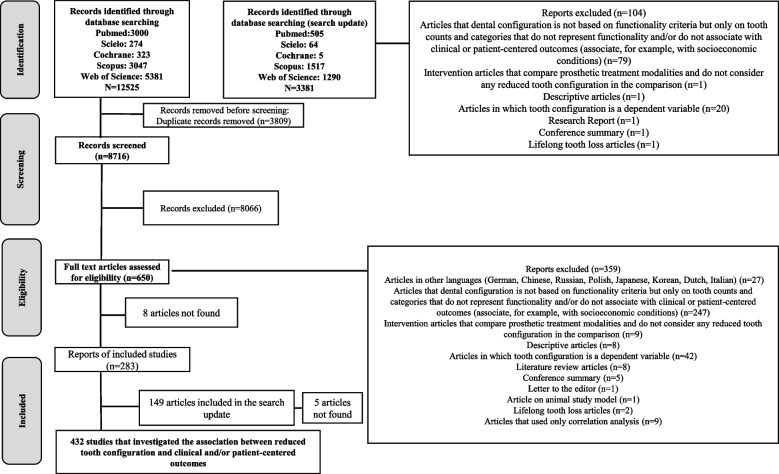
PRISMA flow diagram outlining Search strategy and results along various steps

The searches of the databases led to the retrieval of 12,525 records, 3809 of which were duplicates and were removed, resulting in 8716 records for screening. After the reading of the full texts of 650 articles, 359 were removed and 283 met the inclusion criteria. The updating of the search led to the identification of an additional 149 articles eligible for inclusion in the review (Fig. 1).

Selected articles have been published between 1978 and 2023. An increase in the number of publications was found beginning in 2011. Japan was the country with the largest number of publications (*n* = 136), followed by Brazil (*n* = 39) and the United States (*n* = 29) (see Additional file 2).

Most articles were observational epidemiological studies (*n* = 359; 86.9%), among which the cross-sectional design was the most frequent (*n* = 257; 62.22%). Experimental studies totaled seven articles and the design was not identified in 67 articles (15.50%). A large part of the studies involved samples only of older people (*n* = 244). The others were conducted only with adults (*n* = 28) and 12 studies included adolescents in the sample. The most frequent form of sampling was non-probabilistic (convenience, sample by quotas, consecutive) (*n* = 218). Additional file displays the characteristics of the studies included (see Additional file 3).

The shortened dental configuration based exclusively on tooth count established as a WHO goal was the most frequent (*n* = 206; 49.87%), followed by the count of dental occluding pairs (*n* = 55; 13.31%) and the Eichner Index (*n* = 39; 9.44%). Other configurations reported were the count of functional tooth units (*n* = 31; 7.5%), SDA (*n* = 26; 6.5%), Functional Dentition Classification System (*n* = 10; 2.5%) and other definitions (*n* = 15; 3.75%). Some studies also used combinations of two or three definitions of shortened dental configurations (*n* = 50; 11.57%). The assessment of shortened dental configuration was performed through clinical examinations in most of the studies (*n* = 365). The WHO dental configuration prevailed among the studies over the years (1978–2023). Beginning in 2010, an increase was found in studies that incorporated aspects of functioning, such as occlusion and esthetics, in the assessment of dental configurations (see Additional file 4).

An approximately equal number of articles focused on general health (*n* = 203; 49.1%) and oral health (*n* = 201; 48.6%) as shown in Tables 2, 3, and 4, encompassing both clinical and patient-centered outcomes. Approximately 10% of the total identified studies investigated associations between shortened dental configurations and health behavior (dietary patterns) (*n* = 43; 10.3%) and mortality (*n* = 38; 9.2%) (Table 5). Oral health outcomes were mainly person-centered (*n* = 134; 66.6%). With regards to general health, most studies investigated associations between shortened dental configurations and the presence of diseases/adverse conditions, disabilities or functional loss (*n* = 184; 90.64%), whereas few investigated person-centered measures (*n* = 19; 9.35%).

**Table 2:** General health clinical outcomes classified according to the International Classification of Functioning, Disability and Health and the International Classification of Diseases (ICD) of WHO (*n* = 184)

**International Classification of Functioning, Disability and Health (ICF)**				
**Functionality and Disability (*n* = 65)**	**ICF components**	**ICF Chapters**	**Categories**	**General health clinical outcomes identified in selected studies**
	**Body functions (*n* = 54)**	Mental functions (*n* = 26)	Global mental functions - Intellectual functions (*n* = 24)	• Cognitive function^147,149,159,164,179,212,224,239,258,278,314,320,326,350,382,385,405,411,429^ • Subjective cognitive complaints^369^ • Cognitive impairment^177,243,419^ • Activities of daily living (cognitive domains) ^c288^
			Global mental functions - Sleep functions (*n* = 2)	• Sleep duration^253,294^
			Specific mental functions – Memory functions (*n* = 1)	• Activities of daily living (cognitive domains) ^c288^
		Sensory functions and pain (*n* = 2)	Hearing and vestibular functions (*n* = 2)	• Hearing loss^49,244^
		Functions of the cardiovascular, haematological, immunological and respiratory systems (*n* = 4)	Functions of the cardiovascular system- Heart functions (*n* = 1)	• Heart rate^41^
			Functions of the cardiovascular system - Blood pressure functions (*n* = 4)	• Blood pressure^41^ • Systolic Blood Pressure^133,303^ • Diastolic Blood Pressure^133,303^ • Cardiovascular health^287^
		Functions of the digestive, metabolic and endocrine systems (*n* = 8)	Functions related to the digestive system - Ingestion functions (*n* = 8)	• Oropharyngeal dysphagia^162,255^ • Dysphagia risk^225^ • Complaints about oral-pharyngeal function^15^ • Repetitive Saliva Swallowing Test^84,311^ • Swallowing threshold^138,151^
		Genitourinary and reproductive functions (*n* = 1)	Urinary functions (*n* = 1)	• Kidney function^209^
		Neuromusculoskeletal and movement-related functions (*n* = 13)	Movement functions - Gait pattern functions (*n* = 7)	• Timed 10 m walk test^197^ • Reduction in walking speed^259^ • Mobility disability^80^ • Physical fitness (Maximal stepping rate for 10 seconds)^35,48^ • Gait performance^332^ • Walking speed^286^
			Movement functions - Involuntary movement reaction functions (*n* = 7)	• Physical fitness (one-leg standing time with eyes open)^35,48,64,95,136^ • Standing balance^286^ •Standing Motion^347^
			Muscle functions - Muscle power functions (*n* = 10)	• Handgrip strength^64,136,319^ • Physical fitness (Maximal isometric knee extensor strength)^35^ • Physical fitness (Maximum hand grip strength)^35,48,95,197,263^ • Physical fitness (Maximal leg extensor power)^35,48^ • Physical fitness (Leg extensor strength)^48^ • Chair stands^286^ • Standing Motion^347^
	**Body structures (*n* = 1)**	Structures of the nervous system (*n* = 1)	Structure of brain (*n* = 1)	• Brain structure (volumes of gray matter and white matter)^241^
	**Activities and participation (*n* = 10)**	Communication (*n* = 1)	Conversation and use of communication devices and techniques (*n* = 1)	• Instrumental activities of daily living (making telephone calls)^344,412^
		Mobility (*n* = 6)	Changing and maintaining body position - Transferring oneself (*n* = 5)	• Activities of daily living (as transfer)^a147,195,256,412^ • Activities of daily living^c288^ • Fatigue^d113^
			Walking and moving – Walking (*n* = 6)	• Activities of daily living (mobility)^a28,147,195,256,412^ • Activities of daily living (motor domains) ^c288,412^ • Fatigue^d113^
			Walking and moving - Moving around (*n* = 5)	• Activities of daily living (walking stairs)^a147,195,256,412^ • Activities of daily living (motor domains) ^c288,412^ • Fatigue^d113^
			Walking and moving - Moving around in different locations (*n* = 6)	• Activities of daily living (mobility, toilet use)^a147,195,256,412^ • Activities of daily living (motor domains) c^288,412^ • Homeboundness^199^ • Fatigue^d113^
		Self-care (*n* = 7)	Washing oneself (*n* = 4)	• Activities of daily living (bathing)^a147,195,256,412^ • Activities of daily living (motor domains) ^c288,412^
			Caring for body parts (*n* = 4)	• Activities of daily living (hygiene)^a147,195,256,412^ • Activities of daily living (motor domains) ^c288^
			Toileting (*n* = 4)	• Activities of daily living (controlling bladder)^a147,195,256,412^ • Activities of daily living (controlling bowel)^a147,195,256^ • Activities of daily living (motor domains) ^c288^
			Dressing (*n* = 4)	• Activities of daily living (dressing)^a147,195,256^ • Activities of daily living (motor domains) ^c288^
			Eating (*n* = 4)	• Activities of daily living (feeding)^a147,195,256^ • Activities of daily living (motor domains) ^c288^
			Self-care, unspecified (*n* = 3)	• Instrumental activities of daily living (taking medications)^344^ • Higher level functional capacity^b193,305^
		Domestic life (*n* = 3)	Acquisition of necessities - Acquisition of goods and services (*n* = 3)	• Instrumental activities of daily living (shopping for groceries)^344,412^ • Instrumental activities of daily living (managing money such as paying bills and keeping track of expenses)^344,412^ • Higher level functional capacity^b193,305^
			Household tasks - Preparing meals (*n* = 3)	• Instrumental activities of daily living (preparing a hot meal)^344^ • Higher level functional capacity^b193,305^
			Caring for household objects and assisting others - Caring for household objects (*n* = 3)	• Instrumental activities of daily living (doing work around the house or garden)^344,412^ • Higher level functional capacity^b193,305^
		Interpersonal interactions and relationships (*n* = 2)	General interpersonal interactions - Basic interpersonal interactions (*n* = 2)	• Higher level functional capacity^b^^193,305^
		Community, social and civic life (*n* = 2)	Recreation and leisure (*n* = 2)	• Higher level functional capacity^b193,305^
**International Classification of Diseases (ICD) of WHO (ICD-11)**				
**Hierarchical Classification**				**General health clinical outcomes identified in selected studies**
Neoplasms (*n* = 1)		Malignant neoplasms, except primary neoplasms of lymphoid, haematopoietic, central nervous system or related tissues (*n* = 1)		• Prognosis of patients with colorectal cancer^336^
Diseases of the blood or blood-forming organs(*n* = 1)		Anaemias or other erythrocyte disorders (*n* = 1)		• Anemia^257^
Endocrine, nutritional or metabolic diseases (*n* = 57)		Nutritional disorders (*n* = 51)		• General and central obesity^130,137,182,194,238,266,348^ • Nutritional status (measured by other parameters such as serum albumin dosage, among others)^22,71,79,162,262,268,401^ • Arm circumference^118^ • Waist circumference^300^ • Waist-to-height ratio^348^ • Triceps skinfold^118,300^ • Body Mass Index ^22,39,40,43,64,68,71,79,101,118,134,143,161,180,196,202,262,264,268,271, 279,282,292,298,300,396^ • Weight loss^351^ • Nutritional status (Mini Nutritional Assessment- well nourished, at risk of malnutrition and malnourished/ Council of Nutrition Appetite Questionnaire -CNAQ)^135,141,143,147,152,162,202,288,298,325,329,353,354,368,372,386^ • Prognostic Nutritional Index^317^ • Body composition^378^ • Malnutrition (weight loss, low body mass index, calf Circumference)^e345^
		Metabolic disorders (*n* = 8)		• Systemic inflammatory conditions (concentrations of high-density-lipoprotein cholesterol, low-density-lipoprotein cholesterol, C-reactive protein (CRP) and hemoglobin A1c)^234^ • Metabolic syndrome^185,213,238,261,348,375,424^
Mental, behavioural or neurodevelopmental disorders (*n* = 12)		Neurocognitive disorders (*n* = 7)		• Dementia (onset, incident)^28,131,132,178,334,399^ • Development of all-cause dementia and its subtypes^210^
		Anxiety or fear-related disorders (*n* = 1)		• Anxiety^260^
		Mood disorders (*n* = 5)		• Depression^260,422^ • Depressive symptoms^163,219,371^
Diseases of the n nervous system (*n* = 10)		Disorders with neurocognitive impairment as a major feature (*n* = 1)		• Alzheimer’s disease^352^
		Cerebrovascular diseases (*n* = 7)		• Adverse cardiovascular events (non-fatal myocardial infarction, or non-fatal stroke, non-fatal or fatal MI, non-fatal or fatal stroke)^183,218,359^ • Myocardial infarction^103^ • Cardiovascular risk factors^94^ • Incident hospitalisation for ischaemic heart disease, heart failure, ischaemic stroke and peripheral vascular disease^220^ • Lacunar infarcts^388^
		Symptoms, signs or clinical findings of the nervous system (*n* = 2)		• Multiple falls during the past year^184^ • Risk of falls^242^
Diseases of the visual system (*n* = 2)		Disorders of the eyeball - posterior segment (*n* = 2)		• Diabetes and incident diabetes ^418^ • Diabetic retinopathy^215^
Diseases of the circulatory system (*n* = 6)		Hypertensive diseases (*n* = 2)		• Hypertension^236,398^
		Diseases of arteries or arterioles (*n* = 3)		• Atherosclerosis^216,383^ • Coronary atherosclerotic burden^148^
		Ischaemic heart diseases (*n* = 1)		• Coronary heart disease^29^
Diseases of the respiratory system (*n* = 2)		Certain lower respiratory tract diseases (*n* = 2)		•Incidence of postoperative respiratory complications^432^ •Airflow obstruction^187^
Diseases of the digestive system (*n* = 1)		Diseases of liver (*n* = 1)		• Nonalcoholic fatty liver disease^290^
Diseases of the musculoskeletal system or connective tissue (*n* = 6)		Soft tissue disorders (*n* = 4)		• Sarcopenia^198,217,363,370^ • Non-sarcopenia^363^
		Osteopathies or chondropathies (*n* = 2)		• Metacarpal bone mineral density^56^ • Bone mineral density^64^
Diseases of the genitourinary system (*n* = 2)		Diseases of the urinary system (*n* = 2)		• Chronic kidney disease^214^ • End stage renal disease^328^
Certain conditions originating in the perinatal period (*n* = 1)		Disorders of newborn related to length of gestation or fetal growth (*n* = 1)		• Infant birth weight^289^
Symptoms, signs or clinical findings, not elsewhere classified (*n* = 25)		Symptoms, signs or clinical findings of blood, blood-forming organs, or the immune system (*n* = 5)		• Serum albumin level^105^ • Levels of C-reactive protein^46^ • Malnutrition (Levels of C-reactive protein)^e345^ • Prognostic biomarkers^218^ • Serum lipid peroxide concentration^393^
		Symptoms, signs or clinical findings of the musculoskeletal system (*n* = 3)		• Skeletal muscle mass^181,197^ • Appendicular skeletal muscle mass^240^
		General symptoms, signs or clinical findings (*n* = 2)		• Onset of fever^333^ • Febrile status^93^
		Ageing associated decline in intrinsic capacity (*n* = 20)		• Physical frailty^211,217^ • Frailty^160,235,320,322,355,366,380,384,385,404,415,417,426^ • Incidence of functional disability^281,371^ • Disability^217,415,425^ • Musculoskeletal frailty^221^ • Functional disability^222^
Injury, poisoning or certain other consequences of external causes (*n* = 2)		–		• Fall-related fractures^331,407^

^a^Measured through the Barthel Index

^b^Índice de Competência de Gerontologia do Instituto Metropolitano de Tóquio (TMIG - IC)

^c^Functional independence measure

^d^Avlund Mobility-Tiredness Scale

^e^Global Leadership Initiative on Malnutrition

The same study may present more than one outcome classified into different categories

Full references are described in the Additional file 3

The superscript numbers are the articles selected in the review numbered according to Additional file 3

**Table 3:** Clinical oral health outcomes classified according to the International Classification of Functioning, Disability and Health and the WHO International Classification of Diseases (*n* = 67)

**International Classification of Functioning, Disability and Health (ICF)**				
**Functionality and Disability**	**ICF components**	**ICF Chapters**	**Categories**	**Clinical oral health outcomes identified in selected studies**
	**Body functions (*n* = 42)**	Voice and speech functions (*n* = 1)	Articulation functions (*n* = 1)	• Oral diadochokinesis^346^
		Functions of the digestive, metabolic and endocrine systems (*n* = 44)	Functions related to the digestive system (ingestion functions) (*n* = 44)	• Masticatory performance^1,26,27,66,67,82,97,111,116,125,126,151,167,^ ^226,246,247,295,299, 306,338,340,342,346,347,349,361,378,379,409,420^ • Habitual chewing patterns (chewing strokes, chewing time, mealtime duration, and bite force)^330^ • Mastication predominance^167^ • Salivary flow^84,89,96^ • Salivary microbial^46^ • Occlusal force^151,165,341,347^ • Maximum bite force^18,26,38,51,84,126,268,346^ • Xerostomia^323^ • Hyposalivation^323^ • Tongue pressure^316,346,364^
**International Classification of Diseases (ICD) of WHO (ICD-11)**				
**Hierarchical Classification**				**Clinical oral health outcomes identified in selected studies**
Diseases of the digestive system		Diseases or disorders of orofacial complex (*n* = 24)	Diseases of hard tissues of teeth (*n* = 4)	• Caries experience^75^ • Occlusal stability (occlusal tooth wear)^31,166^ • Attrition^5^
			Periodontal disease (*n* = 5)	• Periodontal disease^8,379^ • Periodontal support^7,31^ • Occlusal stability (alveolar bone support)^11,31^
			Dentofacial anomalies (*n* = 19)	• Migration of the teeth^3^ • Occlusal stability (interdental spacing)^11,31^ • Spaces in anterior teeth^5^ • Occlusal stability (vertical and horizontal overbite)^5,11,31^ • Signs and symptoms of mandibular/craniomandibular dysfunction^4,5,10,58,76,139,223,245,275,427^ • Mobility of the condyles of the temporomandibular joints^140^ • Antegonial index and the mental index^63^ • Temporomandibular joint sounds^25,395,397*^ • Feeling of stiffness or fatigue of the jaws^25,395,397a^ • Difficulty in opening the mouth wide or in locking the mouth^25,395,397a^ • Luxation^25,395,397a^ • Pain on movement^25,395,397a^ • Facial and jaw pain^25,395,397a^ • Occlusal contact^5^ • Occlusal stability (number of occlusal contacts in the anterior region)^11,31^ • Bite stability^237^ • Bruxism^5,25^

^a^Measures used by Índice de Helkimo,1974

Full references are described in the Additional file 3

The superscript numbers are the articles selected in the review numbered according to Additional file 3

**Table 4:** Person-centered outcomes, subjective or person-reported measures related to general health and oral health (*N* = 153)

Outcome classification	Patient-centered outcomes
**Patient-centered outcomes (general aspects of life and health) (*n* = 19)**	• Health-related quality of life^69,87,90,205,270,315,371,389,394^ • Life satisfaction^87,249,371^ • General health status^144,204,206,228,416^ • Self-reported general health^249,276^ • Self-rated happiness^358^ • Sleep quality^297,360^
**Person-centered outcomes (oral health) (*n* = 134)**	• Oral-health-related quality of life^30,34,37,50,52,53,55,57,61,70,86,87,90,99,110,121,122,127,^ ^128,129,145,146,155,156,158,170,171,172,174,175,189,190,191,200,203,204,205,207,^ ^230,231,232,250,270,272,280,296,308,321,335,339,343,349,353,357,374,376,378,381,387,390,402,410^ • Impaired oral-health-related quality of life^120^ • Impacts on daily performance^227,373^ • Chewing ability^5,6,10,12,17,20,23,32,38,45,60,85,88,106,108,109,114,115,123,124,^ ^125,136,142,154,157,165,251,252,268,293,295,308,327,342,349,392,420^ • Self-assessed oral health^100,102,108,119,150,153,229,249,254,276,423^ • Importance of oral health^74^ • Satisfaction with oral status^13,16,24,74,227,389^ • Food-related oral discomfort^324^ • Signs and symptoms of mandibular/craniomandibular dysfunction^4,5,10,12,42,76^ • Reported dental appearance^6,10,108^ • Satisfaction with appearance^74^ • Aesthetics^5,12,92^ • Aesthetics and embarrassment^18^ • Preferred chewing side^91,408^ • Satisfaction with chewing ability^21,77^ • Dissatisfaction with chewing ability^78^ • Perceived needs for dental treatment^313^ • Self-perception of speech^108^ • Speaking difficulty^20^ • Absence of pain or distress^6,10^ • Satisfaction with chewing^74^ • Chewing limitation^18^ • Chewing problems^377^ • Chewing difficulty onset^62,413^ • Functional sociodental impact^18^ • Pain impacts^18^ • Orofacial pain^353^ • Discomfort and other symptoms^18^ • Self-perceived need for complete dentures^362^ • Malnutrition (measured by GOHAI)^a345^

^a^Global Leadership Initiative on Malnutrition

The same study may have addressed centered measures on general health and oral health

Full references are described in the Additional file 3

The superscript numbers are the articles selected in the review numbered according to Additional file 3

**Table 5:** Outcomes related to health behavior (diet patterns) (*n* = 43) and mortality (*n* = 38)

**Health behaviour**	
Health behaviour (diet patterns) (*n* = 43)	• Intake of energy, nutrients, fiber, fruit and vegetables (food diary/questionnaire – 24-h dietary recall/Healthy Eating Index/ Food Frequency Questionnaire)^2,9,14,33,36,38,43,44,54,59,72,98,101,107,117,118,168,169,173,188,201,248,282,283,285,300,301,312,337,365,367,391,414,417,428,431^ • Food consistency^267,307,310^ • Food selectivity behaviour^284^ • Food selection^43,124^ • Food avoidance^15,269^
**Mortality**	
Mortality (*n* = 38)	• Mortality^14,28,46,47,73,80,81,83,104,112,176,183,186,192,208,217,218,220,222,233,265,273,274,277,291,302,304,309,318,345,356,359,400,403,406,421,430^ • Survival rates^65^

Full references are described in the Additional file 3

The superscript numbers are the articles selected in the review numbered according to Additional file 3

Among clinical outcomes of general health, most studies were classified by the body functions component of the ICF, the most addressed chapters of which were mental functions (*n* = 26), neuro-musculoskeletal and movement-related functions (*n* = 13) as well as functions of the digestive, metabolic and endocrine systems (*n* = 8), considering measures of disability and functional impairment. Cognitive functions were frequent outcomes (*n* = 24), as were the capacity to perform and participate in life activities, which regards communication, personal care, domestic life, interpersonal relations and interactions and community, social and civic life (*n* = 10). Endocrine, nutritional or metabolic diseases (*n* = 57), especially nutritional disorders (*n* = 51), were the main general health conditions classified by the ICD-11 (Table 2).

Clinical oral health outcomes were mainly functional aspects, classified as voice and speech functions (*n* = 1) and functions of the digestive, metabolic and endocrine systems (*n* = 42). Diseases or disorders of the orofacial complex were also considered clinical oral health outcomes associated with shortened dental configurations (*n* = 23), the most frequent of which were dentofacial anomalies (*n* = 18) related mainly to temporomandibular disorders and occlusal relationships, followed by periodontal disease (*n* = 5) and diseases of the hard tissues of the teeth (*n* = 4) (Table 3).

Person-centered outcomes related to general life measures or general health were self-perceived general health, happiness, satisfaction with life and general measures of quality of life, the latter of which was assessed using the SF-36, SF-12, EQ-5D, EuroQol, WhoQol-Bref, RAND-36 and OHQoL-UK(W)q©. Person-centered oral health outcomes were measures of OHRQoL (*n* = 62) and chewing ability (*n* = 37). Instruments for assessing OHRQoL were the Oral Health Impact Profile (OHIP) (*n* = 28), Geriatric Oral Health Assessment Index (GOHAI) (*n* = 16) and Oral Impacts on Daily Performance (OIDP) (*n* = 9).

**Fig. 2:**
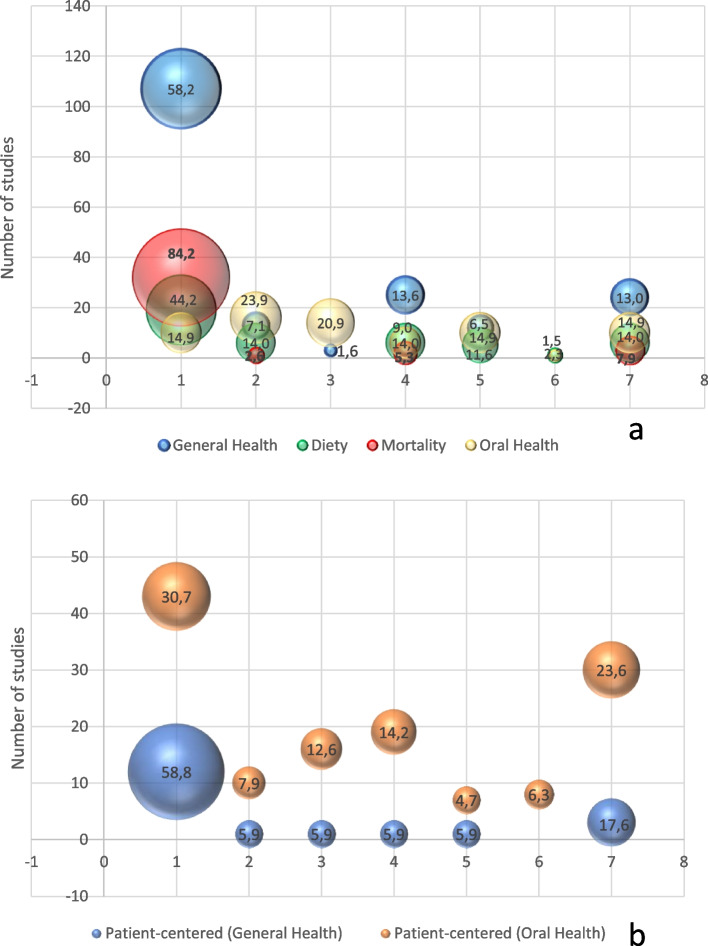
Distribution of studies according to clinical (**a**) and person-centered (**b**) health outcome type and shortened dental configurations (numbers 1 to 7 on the x-axis)

Studies that investigated general health outcomes and mortality more frequently employed the WHO concept of shortened dental configuration, represented by the larger diameter of red and blue circles in Fig. 2a. Among the studies that assessed clinical general health outcomes, 107 (58.15%) employed the WHO classification as exposure. The proportion of studies addressing clinical outcomes of mortality and employed the WHO classification as exposure was 84.21% (*n* = 32). This classification was also the most widely used to investigate associations with person-centered general health (*n* = 12; 63.15%) and oral health (*n* = 43, 32.09%) outcomes. For clinical oral health outcomes, the Eichner Index and SDA were the most employed (*n* = 16 [23.88%] and *n* = 14 [20.89%], respectively). The Functional Dentition Classification System was only used with clinical oral health outcomes, diet and person-centered outcomes (1.49, 2.32 and 5.9%, respectively) (Fig. 2a, b).

*The diameter of the ball represents the percentage of studies that used a given clinical outcome. On the x-axis, values from 1 to 7 correspond to the different shortened dental configurations: 1 = WHO, 2 = Eichner index, 3 = Shortened dental archs, 4 = Dental occluding pairs, 5 = Functional tooth units, 6 = Functional classification system of dentitions, 7 = Other classifications + combinations.

**Fig. 3:**
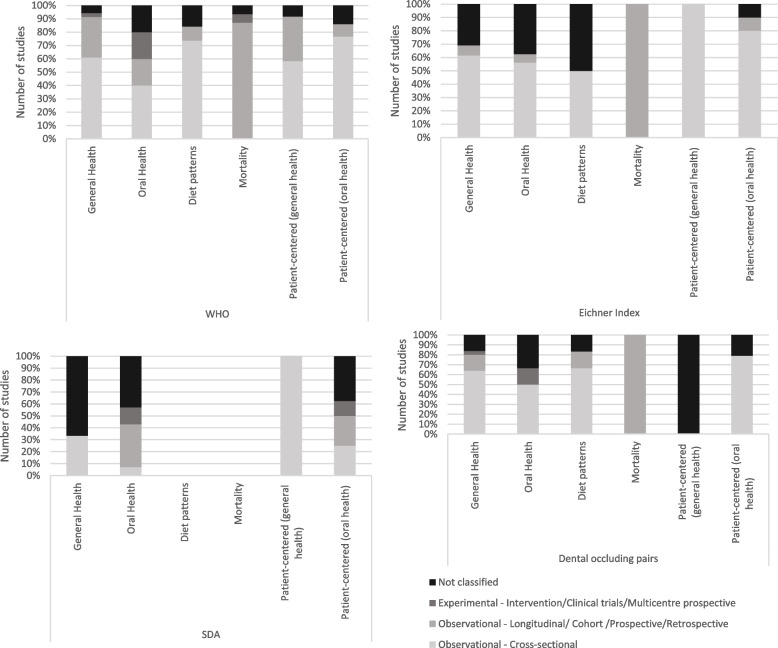
Health outcomes according to the type of study considering each shortened dental configuration (WHO, Eichner index, SDA and Dental occluding pairs)

The cross-sectional study design was the most frequent for investigating associations between most types of shortened dental configurations and all outcomes, except mortality, for which the longitudinal design was the most frequent. A greater diversity of study designs was found in which the WHO configuration was employed. Occluding pairs was the definition employed in case-control studies. Considering the most frequent types of studies, no articles addressed outcomes related to health behavior (dietary patterns) or mortality for the SDA classification (see Additional file 5 and Fig. 3).

More than 80% of the articles described statistically significant associations between the shortened dental configuration studies and health outcomes employing different analytical approaches [see Additional file 6].

## Discussion

The present scoping review demonstrated that shortened dental configurations have been investigated in relation to clinical outcomes of general and oral health. A greater number of studies used person-centered oral health measures as the outcome compared to general health measures, with OHRQoL and chewing ability commonly investigated. Studies using the functional dentition concept based on the number of teeth prevailed for all outcomes. These findings lend strength to multidimensional oral health theoretical models that address functional aspects of oral diseases/adverse conditions and the implications for general health. However, the results indicate that general subjective aspects and wellbeing require greater investigation as outcomes of studies on the effects of shortened dental configurations. With regards to the components of the ICF model considering clinical outcomes of general health, there was a predominance of body functions, activities and participation, representing aspects of clinical-functional conditions, especially in studies involving older people. For clinical oral health outcomes, most were classified as functions of the digestive, metabolic and endocrine systems. Considerable methodological variation was found among the studies; the most prevalent were the cross-sectional design and non-probabilistic sampling.

The dental configuration that considers the 20/21 teeth categories (WHO) was the most employed. The greater ease of collecting the number of teeth in epidemiological studies, which can also be obtained in a valid way through self-reports of the participants, may explain this finding [53, 54]. Discussions are found in the literature on the number of teeth needed to maintain oral functions. Thus, other definitions have been studied taking into account that the mere quantity of teeth is not sufficient to define a dentition model [8, 22]. The present review demonstrates advances in studies incorporating other definitions that consider the position of the teeth [55–57], aspects related to dental occlusion [22, 26, 58] and periodontal status [23]. The challenges of measuring functional aspects of the dentition combining clinical and subjective measures persist in epidemiological research. Studies that validate the use of the number of teeth for the assessment of functional dentition should be conducted in different populations. Moreover, indices that include non-clinical dimensions should be considered [5, 59].

Clinical general health outcomes were the majority among the studies, with a predominance of mental functions, specifically cognitive functions [24], and musculoskeletal functions [60]. The predominance of these outcomes for the shortened dental configuration exposure may be attributed to the fact that the samples were mainly composed of older people, which is an age group with an epidemiological profile of tooth loss and whose functional health and degrees of autonomy and independence are determinants in the aging process more than the presence of disease per se. However, studies that take into account functions of the mouth in the course of life may determine how such functions, together with other determinants, affect the health and illness process. Endocrine, nutritional and metabolic diseases – especially nutritional disorders – were also frequently assessed in relation to shortened dental configurations. Tooth loss is associated with chewing efficiency, which, in turn, can exert an influence on the choice of foods, compromising these health conditions [61]. Dietary patterns are the most often investigated health outcomes. It is important to mention that nutritional aspects constitute the explanatory pathway for associations between oral health and general health and constitute an important outcome in the assessment of functional aspects of oral health [15, 29, 30]. In the present scoping review, this outcome was also the most investigated in studies involving older people. The rapid aging of the world population may be reflected in the increase in publications on dietary patterns beginning in 2019.

With regard to clinical oral health outcomes, ingestion functions were the most addressed, with a predominance of the analysis of chewing performance. This result was expected, as occlusal status can affect chewing efficiency and ingestion outcomes as exposure are parts of the functioning of the stomatognathic system [13, 40, 62]. Other outcomes were highly correlated with tooth loss, such as periodontal disease, occlusal stability, spaces between anterior teeth and the migration of teeth. For such outcomes, shortened dental configurations that consider aspects such as occlusion rather than the mere number of teeth were more common. This may be understood by the characteristics of the studies in which these configurations were employed, a large part with non-probabilistic samples and data collection conduction in a clinical setting, favoring the use of definitions that require more time and resources for the assessment. Moreover, these configurations may have been chosen by authors seeking more sensitive measures of chewing performance, which is affected by the number of teeth in contact with respective antagonists [62]. However, studies on validity and reliability measures of shortened dental configurations remain scarce in the literature [59].

Person-centered outcomes related to oral health were more frequent than clinical oral health outcomes. This difference may be attributed to the growing recognition that normative assessments are insufficient for assessing functional aspects of oral health and their physical and psychosocial impacts on the lives of individuals [11, 12, 28], OHRQoL assessment instruments [63–66] were employed to measure person-centered outcomes, the most widely used of which was the OHIP [67, 68]. However, there is a need to expand the analysis of the effects of shortened dental configurations by adopting more comprehensive theoretical models in which the subjective assessment of oral health composes the assessment of oral functions in the complex determination of the health and wellbeing of individuals [6, 34]. The inclusion of subjective indicators has made valuable contributions to a concept of oral health as an essential component of overall health and wellbeing. However, it should be stressed that OHRQoL instruments measure the physical, psychological and social impacts of oral diseases on the lives of individuals or the performance of activities and are insufficient for assessing the meaning and importance of these impacts on quality of life [69]. Thus, other methodological strategies, such as qualitative studies and the inclusion of quality of life and general health outcomes, should be encouraged to investigate the effects of oral functions as well as produce and consolidate evidence that assumes a more holistic perspective of health. This recommendation is reinforced by the findings of the present review, which found few studies that assessed associations between shortened dental configurations and a more general perception of life, overall quality of life or health-related quality of life [70, 71]. There is also a need to advance in the creation of more comprehensive theoretical models that include oral health as an explanatory factor of general health, quality of life and wellbeing, while also considering individual, social and contextual determinants. Recent discussions on the concept of oral health and the use of the ICF model were important for incorporating the multifaceted nature of the attributes of oral health and the complex interactions between disease and condition status, physiological function and psychosocial function [6]. The ICF provides a theoretical model and operational classification capable of collecting data on the social and environmental contexts of individuals in a multidisciplinary perspective with a focus on functioning rather than the presence/absence of diseases [34].

The effects of dental configurations on mortality have been discussed considering that tooth loss is associated with a reduction in nutrient ingestion due to impaired chewing function, increasing the risk of falls [72], sarcopenia, cognitive decline [73] and the need for nursing care [74]. However, studies need to describe possible biological explanations better to validate these findings [75]. Moreover, researchers point to the need to investigate the possible impact of other factors, such as smoking and access to health services, on the total or partial mediation of the association between oral health status and mortality [76].

Cross-sectional studies were the most frequent for all outcomes. Thus, causal mechanisms are in need of robust evidence based on models of functional health and its determinants [77, 78]. Moreover, epidemiological studies were conducted with non-probabilistic samples and patients recruited from the clinical setting, which limits the external validity of the findings. A considerable diversity was also found in the measures and methods for assessing general and oral health. Thus, classifications such as the ICF could guide the greater standardization of studies, favoring the comparability of findings and the production of more robust evidence [34]. The diversity of health outcomes found in the present scoping review may be the reflection of a discussion that is still in development on what should be measured and how to collect the essential elements of health for the entire population [34].

This study did not include material or studies that were not published in academic periodicals, such as governmental documents and annals of scientific events, which increases the risk of selection bias. However, this decision was necessary due to the large volume of work identified from the strategies adopted. Future systematic reviews should investigate the association between shortened dental configuration and specific outcomes, expanding the search sources.

## Conclusion

Clinical outcomes of general and oral health have been investigated in studies on the effects of shortened dental configurations. The shortened dental configuration defined as a goal by the WHO has been the most discussed. More studies use person-centered measures related to oral health as the outcome compared to those of general health. The findings point to a considerable diversity of health outcomes addressed in the studies selected as well as substantial methodological variability.

### Supplementary Information

**Additional file 1.** Definitions of shortened dental configurations evaluated by the review.

**Additional file 2.** Distribution of studies according to the country of realization. Note: The diameter of the circle represents the frequency of studies at each location.

**Additional file 3 **Characteristics of studies on shortened dental configurations associated with health outcomes (*n*= 432).

**Additional file 4.** Shortened dental configurations addressed in articles published between 1978-2023.

**Additional file 5.** Distribution of types of studies according to shortened dental configurations and analyzed health outcomes.

**Additional file 6.** Statistical analysis used and presence or absence of adjustment for covariates considering clinical health and/or person-centered outcomes.

## Data Availability

All data generated or analysed during this study are included in this published article [and its supplementary information files].

## Abbreviations

WHO: World Health Organization
QoL: quality of life
OHRQoL: oral-health-related quality of life
FD: functional dentition
SDA: Shortened dental arches
POPs: posterior occluding pairs
ICF: International Classification of Functioning, Disability and Health
PRISMA-ScR: Preferred Reporting Items for Systematic Reviews and Meta-Analyses for Scoping Reviews
ICD-11: International Classification of Diseases
BMI: body mass index
GOHAI: Geriatric Oral Health Assessment Index
OIDP: Oral Impacts on Daily Performance

## References

[1] Slade G (2012). Oral health-related quality of life is important for patients, but what about populations?. Community Dent Oral Epidemiol.

[2] Baâdoudi F, Trescher A, Duijster D, Maskrey N, Gabel F, van der Heijden BGJMG (2017). A consensus-based set of measures for oral health care. J Dent Res.

[3] Lee JY, Watt RG, Williams DM, Giannobile WV (2017). A new definition for oral health: implications for clinical practice, policy and research. J Dent Res.

[4] Righolt AJ, Sidorenkov G, Faggion CM, Listl S, Duijster D (2019). Quality measures for dental care: a systematic review. Community Dent Oral Epidemiol.

[5] Ni Riordain R, Glick M, Al Mashhadani SSA, Aravamudhan K, Barrow J, Cole D (2021). Developing a standard set of patient-centred outcomes for adult oral health –an international, cross-disciplinary consensus. Int Dent J.

[6] Glick M, Williams DM, Kleinman DV, Vujicic M, Watt RG, Weyant RJ (2017). A new definition for oral health developed by the FDI world dental federation opens the door to a universal definition of oral health. [editorial]. J Public Health Dent.

[7] CIF: Classificação Internacional de Funcionalidade, Incapacidade e Saúde/[Centro Colaborador da Organização Mundial da Saúde para a Família de Classificações Internacionais em Português, org.; coordenação da tradução Cássia Maria Buchalla]. – 1. ed., 1. reimpre. – São Paulo: Editora da Universidade de São Paulo, 2008.

[8] Gerritsen AE, Allen FP, Witter DJ, Bronkhorst EM, Creugers NHJ (2010). Tooth loss and oral health-related quality of life: a systematic review and meta-analysis. Health Qual Life Outcomes.

[9] Damyanov ND, Witter DJ, Bronkhorst EM, Creugers NH (2013). Satisfaction with the dentition related to dental functional status and tooth replacement in an adult Bulgarian population: a cross-sectional study. Clin Oral Investig.

[10] Tan H, Peres KG, Peres MA (2016). Retention of teeth and oral health-related quality of life. J Dent Res.

[11] Antunes JL, Tan H, Peres KG, Peres MA (2016). Impact of shortened dental arches on oral health-related quality of life. J Oral Rehabil.

[12] Ferreira RC, Kawachi I, Souza JGS, Campos FL, Chalub LLFH, Antunes JLF (2019). Is reduced dentition with and without dental prosthesis associate with oral health-related quality of life? A cross-sectional study. Health Qual Life Outcomes.

[13] Shao Z, Guo X, Zhang Q, Bronkhorst EM, Zou D, Creugers NHJ (2018). Masticatory efficiency in patients with partially dentate dentitions. J Dent.

[14] Kim EJ, Jin BH (2018). Comparison of oral health status and daily nutrient intake between elders who live alone and elders who live with family: based on the Korean National Health and nutrition examination survey (KNHANES VI) (2013-2015). Gerodontology.

[15] Zhang Q, Niesten D, Bronkhorst EM, Witter DJ, Creugers NHJ (2020). Food avoidance is associated with reduced dentitions and edentulousness. Clin Oral Investig.

[16] Käyser AF (1981). Shortened dental arches and oral function. J Oral Rehabil.

[17] World Health Organization (1982). A review of current recommendations for the organization and administration of community Oral health services in northern and Western Europe: report on a WHO workshop: Oslo 24–28 may 1982.

[18] Hobdell M, Petersen PE, Clarkson J, Johnson N (2003). Global goals for oral health 2020. Int Dent J.

[19] Lin HC, Corbet EF, Lo EC, Zhang HG (2001). Tooth loss, occluding pairs, and prosthetic status of Chinese adults. J Dent Res.

[20] Eichner K (1955). Über eine Gruppeneinteilung der Lückengebisse für die Prothetik. Dtsch Zahnarztl Z.

[21] Ikebe K, Matsuda K, Murai S, Maeda Y, Nokubi T (2010). Validation of the Eichner index in relation to occlusal force and masticatory performance. Int J Prosthodont.

[22] Nguyen TC, Witter DJ, Bronkhorst EM, Pham LH, Creugers NHJ (2011). Dental function status in a southern Vietnamese adult population – an analysis by a combined quantitative and qualitative classification system. Int J Prosthodont.

[23] Chalub LLFH, Ferreira RC, Vargas AMD (2016). Functional, esthetical, and periodontal determination of the dentition in 35- to 44-year-old Brazilian adults. Clin Oral Investig.

[24] Zhang XM, Wu X, Chen W (2022). The association between number of teeth and cognitive frailty in older adults: a cross-sectional study. J Nutr Health Aging.

[25] Zhang J, Xu L (2022). Frailty and associated factors among Chinese centenarians. J Nutr Health Aging.

[26] Uy SNMR, Deng K, Fok CTC, Fok MR, Pelekos G, Tonetti MS (2022). Food intake, masticatory function, tooth mobility, loss of posterior support, and diminished quality of life are associated with more advanced periodontitis stage diagnosis. J Clin Periodontol.

[27] Chen HH, Lin PY, Lin CK, Lin PY, Chi LY (2022). Effects of oral exercise on tongue pressure in Taiwanese older adults in community day care centers. J Dent Sci.

[28] Chalub LLFH, Ferreira RC, Vargas AMD (2017). Influence of functional dentition on satisfaction with oral health and impacts on daily performance among Brazilian adults: a population-based cross-sectional study. BMC Oral Health.

[29] Mendonça DD, Furtado MV, Sarmento RA, Nicoletto BB, Souza GC, Wagner TP (2019). Periodontitis and tooth loss have negative impact on dietary intake: a cross-sectional study with stable coronary artery disease patients. J Periodontol.

[30] Logan D, McEvoy CT, McKenna G, Kee F, Linden G, Woodside JV (2020). Association between oral health status and future dietary intake and diet quality in older men: the PRIME study. J Dent.

[31] Osterberg T, Carlsson GE, Sundh V, Mellström D (2008). Number of teeth–a predictor of mortality in 70-year-old subjects. Community Dent Oral Epidemiol.

[32] Hirotomi T, Yoshihara A, Ogawa H, Miyazaki H (2015). Number of teeth and 5-year mortality in an elderly population. Community Dent Oral Epidemiol.

[33] Goto Y, Wada K, Uji T, Koda S, Mizuta F, Yamakawa M, Nagata C (2020). Number of teeth and all-cause and cancer mortality in a Japanese community: the Takayama study. J Epidemiol.

[34] Faulks D, Scambler S, Daly B, Jamieson L, Hennequin M, Tsakos G (2022). Measuring oral health-how can the international classification of functioning help?. Community Dent Oral Epidemiol.

[35] Arksey H, O’Malley L (2005). Scoping studies: towards a methodological framework. Int J Soc Res Methodol.

[36] Peters MD, Godfrey CM, Khalil H, McInerney P, Parker D, Soares CB (2015). Guidance for conducting systematic scoping reviews. Int J Evid Based Healthc.

[37] Tricco AC, Lillie E, Zarin W, O’Brien KK, Colquhoun H, Levac D (2018). PRISMA extension for scoping reviews (PRISMA-ScR): checklist and explanation. Ann Intern Med.

[38] Witter DJ, van Palenstein Helderman WH, Creugers NH, Kayser AF (1999). The shortened dental arch concept and its implications for oral health care. Community Dent Oral Epidemiol.

[39] Elias AC, Sheiham A (1998). The relationship between satisfaction with mouth and number and position of teeth. J Oral Rehabil.

[40] Nguyen TC, Witter DJ, Bronkhorst EM, Gerritsen AE, Creugers NH (2011). Chewing ability and dental functional status. Int J Prosthodont.

[41] Zhang Q, de Witter DJ, Gerritsen AE, de Bronkhorst EM, Creugers NH (2013). Functional dental status and oral health-related quality of life in an over 40 years old Chinese population. Clin Oral Investig.

[42] Zhang Q, Witter DJ, Bronkhorst EM, Creugers NH (2013). Chewing ability in an urban and rural population over 40 years in Shandong Province, China. Clin Oral Investig.

[43] Konishi C, Hakuta C, Ueno M, Shinada K, Wright FA, Kawaguchi Y (2010). Factors associated with self-assessed oral health in the Japanese independent elderly. Gerodontology.

[44] Barbe AG, Javadian S, Rott T, Scharfenberg I, Deutscher HCD, Noack MJ (2020). Objective masticatory efficiency and subjective quality of masticatory function among patients with periodontal disease. J Clin Periodontol.

[45] Zeng X, Sheiham A, Tsakos G (2008). Relationship between clinical dental status and eating difficulty in an old Chinese population. J Oral Rehabil.

[46] Shao R, Hu T, Zhong YS, Li X, Gao YB, Wang YF (2018). Socio-demographic factors, dental status and health-related behaviors associated with geriatric oral health-related quality of life in southwestern China. Health Qual Life Outcomes.

[47] Batista MJ, Lawrence HP, de Sousa ML (2014). Impact of tooth loss related to number and position on oral health quality of life among adults. Health Qual Life Outcomes.

[48] Appollonio I, Carabellese C, Frattola A, Trabucchi M (1997). Influence of dental status on dietary intake and survival in community-dwelling elderly subjects. Age Ageing.

[49] Iwashita H, Tsukiyama Y, Kori H, Kuwatsuru R, Yamasaki Y, Koyano K (2014). Comparative cross-sectional study of masticatory performance and mastication predominance for patients with missing posterior teeth. J Prosthodont Res.

[50] World Health Organization. ICD-11 for mortality and morbidity statistics. Version: 2019 April. Geneva: WHO; 2019 [cited 2022 May 29]. Available from: https://icd.who.int/browse11/l-m/en. Accessed 3 Jan 2022.

[51] Schimmel M, Christou P, Herrmann F, Müller F (2007). A two-colour chewing gum test for masticatory efficiency: development of different assessment methods. J Oral Rehabil.

[52] Arce-Tumbay J, Sanchez-Ayala A, Sotto-Maior BS, Senna PM, Campanha NH (2011). Mastication in subjects with extremely shortened dental arches rehabilitated with removable partial dentures. Int J Prosthodont.

[53] Similä T, Nieminen P, Virtanen JI (2018). Validity of self-reported number of teeth in middle-aged Finnish adults: the northern Finland birth cohort study 1966. BMC Oral Health.

[54] Sekundo C, Stock C, Jürges H, Listl S (2019). Patients' self-reported measures of oral health-a validation study on basis of oral health questions used in a large multi-country survey for populations aged 50. Gerodontology.

[55] Steele JG, Ayatollahi SM, Walls AW, Murray JJ (1997). Clinical factors related to reported satisfaction with oral function amongst dentate older adults in England. Community Dent Oral Epidemiol.

[56] Omar R, Al-Boaijan E, Al-Twaijri S, Akeel R (2007). Satisfaction with oral status among adult school-attending Saudi women with and without posterior fixed partial dentures. Quintessence Int.

[57] Azevedo MS, Correa MB, Azevedo JS, Demarco FF (2015). Dental prosthesis use and/or need impacting the oral health-related quality of life in Brazilian adults and elders: results from a National Survey. J Dent.

[58] van der Heijden EM, Klüter WJ, van der Maarel-Wierink CD, Gobbens RJJ (2022). Exploring associations between multidimensional frailty and oral health in community-dwelling older people. A pilot study. Spec Care Dentist.

[59] Campos FL, Rhodes GAC, Vasconcellos WA, Bomfim RA, Sampaio AA, Chalub LLFH, et al. Validation of pairs of antagonist teeth for the evaluation of shortened dental arch in epidemiological studies. Braz Oral Res. 2023:37:e045. 10.1590/1807-3107bor-2023.vol37.0045 37162058

[60] Shin HS (2019). Handgrip strength and the number of teeth among Korean population. J Periodontol.

[61] Östberg AL, Bengtsson C, Lissner L, Hakeberg M (2012). Oral health and obesity indicators. BMC Oral Health.

[62] Indrasari M, Dewi RS, Rizqi AA (2018). The influence of the number of functional tooth units (FTUs) on masticatory performance. J Int Dent Med Res.

[63] Leao A, Sheiham A (1996). The development of a socio-dental measure of dental impacts on daily living. Community Dent Health.

[64] Slade GD, Spencer AJ (1994). Development and evaluation of the Oral health impact profile. Community Dent Health.

[65] Slade GD (1997). Derivation and validation of a short-form Oral health impact profile. Community Dent Oral Epidemiol.

[66] Adulyanon S (1996). An integrated socio-dental approach to dental treatment need estimation.

[67] Goh V, Nihalani D, Yeung KWS, Corbet EF, Leung WK (2018). Moderate- to long-term therapeutic outcomes of treated aggressive periodontitis patients without regular supportive care. J Periodontal Res.

[68] Bhat M, Bhat S, Brondani M, Mejia GC, Pradhan A, Roberts-Thomson K (2021). Prevalence, extent, and severity of Oral health impacts among adults in rural Karnataka, India. JDR Clin Trans Res.

[69] Locker D, Allen F (2007). What do measures of 'oral health-related quality of life' measure?. Community Dent Oral Epidemiol.

[70] Haag DG, Peres KG, Brennan DS (2017). Tooth loss and general quality of life in dentate adults from southern Brazil. Qual Life Res.

[71] Brennan DS, Mittinty MM, Jamieson L (2019). Psychosocial factors and self-reported transitions in oral and general health. Eur J Oral Sci.

[72] Yamamoto T, Kondo K, Misawa J, Hirai H, Nakade M, Aida J (2012). Dental status and incident falls among older Japanese: a prospective cohort study. BMJ Open.

[73] Yamamoto T, Kondo K, Hirai H, Nakade M, Aida J, Hirata Y (2012). Association between self-reported dental health status and onset of dementia: a 4-year prospective cohort study of older Japanese adults from the Aichi Gerontological evaluation study (AGES) project. Psychosom Med.

[74] Aida J, Kondo K, Hirai H, Nakade M, Yamamoto T, Hanibuchi T (2012). Association between dental status and incident disability in an older Japanese population. J Am Geriatr Soc.

[75] Yu YH, Cheung WS, Steffensen B, Miller DR (2021). Number of teeth is associated with all-cause and disease-specific mortality. BMC Oral Health.

[76] Koka S, Gupta A (2018). Association between missing tooth count and mortality: a systematic review. J Prosthodont Res.

[77] Jockusch J, Hopfenmüller W, Nitschke I (2021). Chewing function and related parameters as a function of the degree of dementia: is there a link between the brain and the mouth?. J Oral Rehabil.

[78] Stewart R, Stenman U, Hakeberg M, Hägglin C, Gustafson D, Skoog I (2015). Associations between oral health and risk of dementia in a 37-year follow-up study: the prospective population study of women in Gothenburg. J Am Geriatr Soc.

