# Supplementary material for: Association between shortened dental configurations and health outcomes: a scoping review
Source: BMC Oral Health. 2024 Jan 19;24:111. doi: 10.1186/s12903-023-03714-4 (PMC10799365; doi:10.1186/s12903-023-03714-4)
Supplement: Supplementary file 1 — Additional file 1. Definitions of shortened dental configurations evaluated by the review. [file 12903_2023_3714_MOESM1_ESM.docx]

**Additional file 1 -** Definitions of shortened dental configurations evaluated by the review

| **Shortened dental configurations** | **Definition** |
| --- | --- |
| Cutoff point of 20/21 teeth based on the WHO | It is based on the “lifetime retention of 20 functional natural teeth without resorting to the use of a prosthesis” and has been adopted by the WHO as part of its oral health goals for developing countries. In 2003, the document “Global goals for oral health 2020” presented the goal of increasing the percentage of individuals with PD with 21 or more teeth (Hobdell *et al.,* 2003) |
| Eichner index | It is based on the presence of natural occlusal contact in the premolar and molar regions. These regions are divided into four support zones, two in the molar region and two in the premolar region. The index divides the occlusal state into three main groups (A, B and C), which are divided into subgroups (A1–A3; B1–B4; C1–C3). Group A: Occlusal contacts are present in all occlusal support zones (Group A1: No missing teeth in the mandible and maxilla; Group A2: At least one missing tooth in the mandible or maxilla; Group A3: At least one missing tooth in the mandible and in the maxilla); Group B: Occlusal contacts are present in three to one contact zone(s) or in the anterior region only (Groups B1, B2, and B3: Posterior occlusal contact(s) in three, two, and one zone(s) , respectively; Group B4: Occlusal contact(s) only in the anterior region); Group C: No occlusal contact (Group C1: At least one tooth in the mandible and maxilla without any occlusal contact; Group C2: At least one tooth in the mandible or maxilla; Group C3: Completely edentulous in both arches) (Eichner, 1955) |
| Shortened Dental Arch (SDA) | It presupposes a dentition with the preservation of 10 teeth, six anteriors and four premolars in each arch (Käyser, 1981). It is defined as keeping the anterior teeth intact plus a functional number of occlusal contacts between the posterior teeth (Witter *et al.,* 1999) |
| Functional Dentition Classification System | It contemplates the criteria of occlusion and aesthetics and is composed of five levels based on the functionality of oral health. The number of natural teeth, types of teeth present and pairs of posterior occlusion are used as evaluation criteria (Nguyen *et al.,* 2011). The first level in the classification (level I) presupposes the presence of at least one natural tooth in the mandible and maxilla (cutoff point: 1 tooth in each arch). The second level (level II) is based on the concept of “20 well-distributed teeth” and the assumption that for adequate oral function it is necessary to distribute at least 10 teeth in each arch to allow for 9 to 10 pairs of opposing teeth (point cutting edge: 10 teeth in each arch) (Elias; Sheiham, 1998). The third level (level III) is based on the assumption that a complete anterior region must be present for esthetic and psychofunctional well-being (cutoff: 12 anterior teeth) (Damyanov *et al.,* 2013; Nguyen *et al.,* 2011b; Zhang *et al.,* 2013). Level IV describes the region of the premolars, which presupposes sufficient oral function if 3 or 4 POPs of premolars are present (cutoff: 3 POPs of premolars) (Nguyen *et al.,* 2011b; Zhang *et al.,* 2013). The fifth level (level V) is based on the recognized but relatively low impact of molars (Nguyen *et al.,* 2011b; Zhang *et al.,* 2013). Faced with the need to make the concept of functional dentition more comprehensive, periodontal evaluation was incorporated into this system. Thus, a sixth level was added, which considered as a favorable periodontal condition the presence of all sextants with a Community Periodontal Index (CPI) score ≤ 3 and Clinical Attachment Loss (PIC) ≤ 1 (Chalub *et al.,* 2016) |
| Count of functional tooth units (FTUs) | These are molar or premolar units. For this classification, a score of one (1) was assigned for each pair of FTU of premolars; and a score of two (2) for each molar FTU |
| Count of dental occluding pairs | Assessed by the presence of posterior and/or anterior dental elements |
| Other definitions | Dental configurations that considered specific types of teeth present or missing (anterior and/or posterior) (Batista *et al.,* 2014), which defined, for example, the presence of 10 upper and six lower teeth) (Apollonio *et al.,* 1997) or that were based on the unilateral or bilateral absence of posterior teeth (Iwashita *et al.,* 2014) |

Note: the same study may have used more than one definition of reduced tooth configuration (for example, WHO and Count of dental occluding pairs)
