# Supplementary figures and images for: Association between shortened dental configurations and health outcomes: a scoping review
Source: BMC Oral Health. 2024 Jan 19;24:111. doi: 10.1186/s12903-023-03714-4 (PMC10799365; doi:10.1186/s12903-023-03714-4)

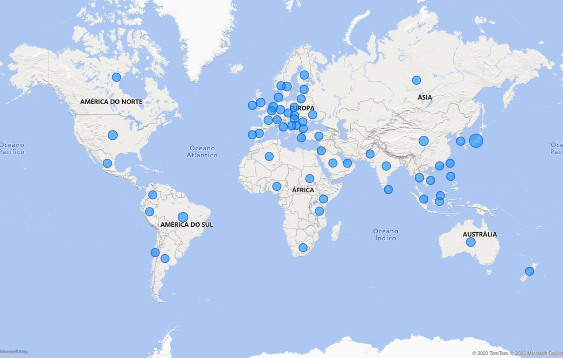

Supplement: Supplementary file 2 — Additional file 2. Distribution of studies according to the country of realization. Note: The diameter of the circle represents the frequency of studies at each location. [file 12903_2023_3714_MOESM2_ESM.tif]

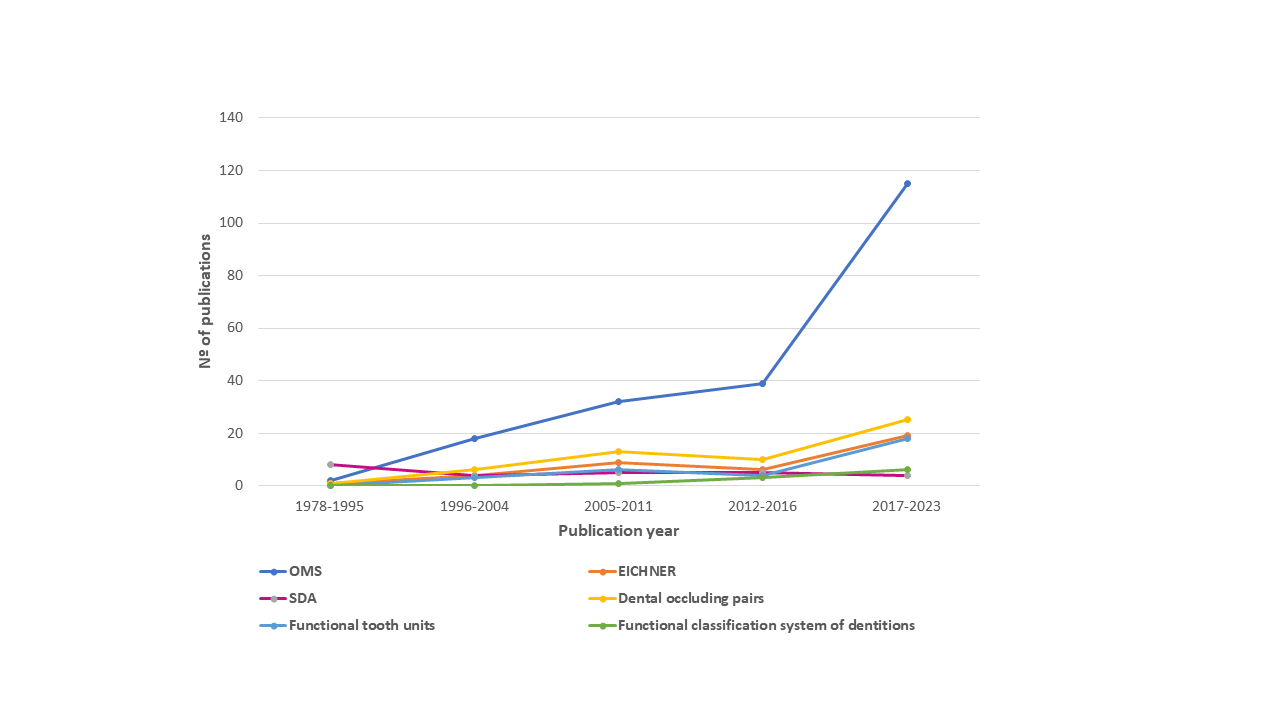

Supplement: Supplementary file 4 — Additional file 4. Shortened dental configurations addressed in articles published between 1978-2023. [file 12903_2023_3714_MOESM4_ESM.tif]
