# Supplementary material for: Association between shortened dental configurations and health outcomes: a scoping review
Source: BMC Oral Health. 2024 Jan 19;24:111. doi: 10.1186/s12903-023-03714-4 (PMC10799365; doi:10.1186/s12903-023-03714-4)
Supplement: Supplementary file 5 — Additional file 5. Distribution of types of studies according to shortened dental configurations and analyzed health outcomes. [file 12903_2023_3714_MOESM5_ESM.docx]

**Additional file 5 -** Distribution of types of studies according to shortened dental configurations and analyzed health outcomes

| **Types of studies/Shortened dental configurations** | ***Observational- Cross-sectional*** | ***Obervational - Longitudinal/*** ***Cohort /Prospective/Retrospective*** | ***Observational- Case- control*** | ***Obervational - Longitudinal and Cross-sectional*** | ***Experimental - Intervention/Clinical trials/Multicentre prospective*** | ***Not Classified*** | | ***Total*** |
| --- | --- | --- | --- | --- | --- | --- | --- | --- |
|  | **General health clinical outcomes (n=184)** | | | | | | | |
| WHO | **64 (34,78%)** | 31(16,85%) | 2(1.09%) | 1(0.54%) | 3(1.63%) | | 6 (3,26%%) | **107(58,15%)** |
| Eichner index | 8(4.35%) | 1(0.54%) | 0 | 0 | 0 | | 4(2.17%) | 13(7.06%) |
| Shortened dental arches | 1(0.59%) | 0 | 0 | 0 | 0 | | 2(1.18%) | 3(1.78%) |
| Dental occluding pairs | 16(8.69%) | 4(2,17%) | 1(0.54%) | 0 | 0 | | 4(2.17%) | 25(13,59%) |
| Functional tooth units | 9(4,89%) | 2(1.09%) | 1(0.54%) | 0 | 0 | | 0 | 12(6,52%) |
| Other classifications + WHO and others + WHO and Dental occluding pairs + WHO and Eichner index + WHO and Functional tooth units | 17(9,24%) | 6(3.26%) | 0 | 0 | 0 | | 1(0.54%) | 24(13,04%) |
| ***Total*** | **115(62.5%)** | **44(23.91%)** | 4(2.17%) | 1(0.54%) | 3(1.63%) | | 17(9.24%) | 184 (100%) |
| **Types of studies/Shortened dental configurations** | **Clinical oral health outcomes (n=67)** | | | | | | | |
| WHO | 4 (5,97%) | 2(2,98%) | 0 | 0 | 2(2,98%) | | 2(2,98%) | 10(14,92%) |
| Eichner index | **9(13.43%)** | 1(1,49%) | 0 | 0 | 0 | | **6(8,95%)** | **16(23.88%)** |
| Shortened dental arches | 1(1.49%) | **5(7.46%)** | 0 | 0 | 2(2,98%) | | 6(8,95%) | **14(20,89%)** |
| Dental occluding pairs | 3(4.48%) | 0 | 1(1.49%) | 0 | 0 | | 2(2,98%) | 6(8,95%) |
| Functional tooth units | **7(10.45%)** | 1(1.49%) | 0 | 0 | 0 | | 2(2,98%) | 10(14,92%) |
| Functional classification system of dentitions | 0 | 0 | 0 | 0 | 0 | | 1(1.49%) | 1(1.49%) |
| Other classifications + Eichner index and Functional tooth units + Shortened dental arches and Dental occluding pairs + WHO and Dental occluding pairs + WHO and Eichner index | **7(10.45%)** | 1(1.49%) | 0 | 0 | 0 | | 2(2,98%) | 10 (14,92%) |
| ***Total*** | **31(46,27)** | 10(14,92%) | 1(1.49%) | 0 | 4(5,97%) | | 21(31,34%) | 67(100%) |
| **Types of studies/Shortened dental configurations** | **Person-centered outcomes (general life or general health) (n=19)** | | | | | | | |
| WHO | **7(36,84%%)** | 4(21,05%) | 0 | 0 | 0 | | 1(5.26%) | **12(63,16%)** |
| Eichner index | 1(5.26%) | 0 | 0 | 0 | 0 | | 0 | 1(5.26%) |
| Shortened dental arches | 1(5.26%) | 0 | 0 | 0 | 0 | | 0 | 1(5.26%) |
| Dental occluding pairs | 0 | 0 | 0 | 0 | 0 | | 1(5.26%) | 1(5.26%) |
| Functional tooth units | 1(5.26%) | 0 | 0 | 0 | 0 | | 0 | 1(5.26%) |
| Other classifications + WHO and Shortened dental arches | 1(5.26%) | 0 | 0 | 0 | 0 | | 2(10,53%) | 3(15,79%) |
| ***Total*** | **11(57,89%)** | 4(21,05%) | 0 | 0 | 0 | | 4(21,05%) | 19(100%) |
| **Types of studies/Shortened dental configurations** | **Person-centered outcomes (oral health) (n=134)** | | | | | | | |
| WHO | **33(24,63%)** | 4(2.98%) | 0 | 0 | 0 | | 6**(4,48%)** | **43(32,09%)** |
| Eichner index | 8(5,97%) | 1(0.75%) | 0 | 0 | 0 | | 1(0.75%) | 10(7.46%) |
| Shortened dental arches | 4(2.98%) | 4(2,98%) | 0 | 0 | 2(1.49%) | | 6**(4.48%)** | 16(11,94%) |
| Dental occluding pairs | 15(11,19%) | 0 | 0 | 0 | 0 | | 4(2,98%) | 19(14,18%) |
| Functional tooth units | 4(2,98%) | 0 | 0 | 0 | 0 | | 3(2,24%) | **7(5,22%)** |
| Functional classification system of dentitions | **6(4.72%)** | 0 | 0 | 0 | 0 | | 2(1.58%) | 8(5,97%) |
| Other classifications + WHO, Dental occluding pairs and others: distribution of teeth + WHO and Dental occluding pairs + Eichner index and Dental occluding pairs + WHO and Dental occluding pairs + WHO and functional tooth units + Eichner index and Functional tooth units + WHO and Eichner index + Shortened dental arches and Functional classification system of dentitions and WHO + Dental occluding pairs and anterior teeth | 21(15,67%) | 2(1.49%) | 0 | 1(0.0,75%) | 0 | | 7(5.22%) | 31(23,13%) |
| ***Total*** | **91(67.91%)** | 11(8.20%) | 0 | 1(0.75%) | 2(1.49%) | | 29(21,64%) | 134(100%) |
| **Types of studies/Shortened dental configurations** | **Outcomes related to health behavior (dietary patterns) (n=43)** | | | | | | | |
| WHO | **14(32,56%)** | 2(4,65%) | 0 | 0 | 0 | | 3(6,98%) | 19(44,19%) |
| Eichner index | 3(6,98%) | 0 | 0 | 0 | 0 | | 3(6,98%) | 6(13,95%) |
| Dental occluding pairs | 4(9,30%) | 1(2.32%) | 0 | 0 | 0 | | 1(2.32%) | 6(13,95%) |
| Functional tooth units | 2(4,65%) | 2(4,65%) | 0 | 0 | 0 | | 1(2.32%) | 5(11,63%) |
| Functional classification system of dentitions | 0 | 0 | 0 | 0 | 0 | | 1(2.32%) | 1(2.32%) |
| Other classifications + WHO and Dental occluding pairs + Dental occluding pairs and others | 4(9,30%) | 2(4,65%) | 0 | 0 | 0 | | 0 | 6(13,95%) |
| ***Total*** | **27(62,79%)** | **7( 16,28%)** | 0 | 0 | 0 | | 9(20,93%) | 43(100%) |
| **Types of studies/Shortened dental configurations** | **Mortality (n=38)** | | | | | | | |
| WHO | 0 | **28(73.68%)** | 0 | 0 | 2**(5.26%)** | | 2(5,26%) | **32(84.21%)** |
| Eichner index | 0 | **1**(2.63%) | 0 | 0 | 0 | | 0 | 1(2.63%) |
| Dental occluding pairs | 0 | **2(5.26%)** | 0 | 0 | 0 | | 0 | 2**(5.26%)** |
| Other classifications + WHO and Dental occluding pairs + WHO and Eichner index | 1(2.63%) | 2**(5.26%)** | 0 | 0 | 0 | | 0 | 3(7,89%) |
| ***Total*** | 1(2.63%) | **33(86,84%)** | 0 | 0 | 2**(5.26%)** | | 2(5,26%) | 38(100%) |

Note: The same study may present more than 1 outcome and, therefore, may be classified in more than one category
