# Supplementary material for: Association between shortened dental configurations and health outcomes: a scoping review
Source: BMC Oral Health. 2024 Jan 19;24:111. doi: 10.1186/s12903-023-03714-4 (PMC10799365; doi:10.1186/s12903-023-03714-4)
Supplement: Supplementary file 6 — Additional file 6. Statistical analysis used and presence or absence of adjustment for covariates considering clinical health and/or person-centered outcomes. [file 12903_2023_3714_MOESM6_ESM.docx]

**Additional file 6-** Statistical analysis used and presence or absence of adjustment for covariates considering clinical health and/or person-centered outcomes

| **Classification of outcomes** | **Statistical analysis performed** | | | | **Strategy to control confounding (stratification, multiple analysis)** | |
| --- | --- | --- | --- | --- | --- | --- |
|  | **Comparison of groups** | **Analysis of variance/covariance** | **Regression Analysis** | **Mediation analysis** | **Yes** | **Not** |
| **General health clinical outcomes (n=184)** | 25 | 12 | **142** | 5 | **145** | 39 |
| **Clinical oral health outcomes (n=67)** | 26 | 8 | **31** | 2 | **28** | 39 |
| **Person-centered outcomes (general life or general health) (n=19)** | 1 | 2 | **16** | 0 | **16** | 3 |
| **Person-centered outcomes (oral health) (n=134)** | 25 | 16 | **89** | 4 | **91** | 43 |
| **Outcomes related to health behavior (dietary patterns) (n=43)** | 8 | 5 | **30** | 0 | **34** | 9 |
| **Mortality (n=38)** | 2 | 0 | **34** | 2 | **36** | 2 |

If a study used group comparison analysis, ANOVA and regression, the result computed was the regression.
